# Acromioclavicular joint suture button repair leads to coracoclavicular tunnel widening

**DOI:** 10.1007/s00167-022-06929-0

**Published:** 2022-03-22

**Authors:** D. Dalos, G. Huber, Y. Wichern, K. Sellenschloh, K. Püschel, K. Mader, M. M. Morlock, K. H. Frosch, T. O. Klatte

**Affiliations:** 1 Department of Trauma and Orthopedic Surgery, University Medical Center Hamburg-Eppendorf, Hamburg, Germany; 2 UKE Athleticum-Center for Athletic Medicine, University Medical Center Hamburg-Eppendorf, Hamburg, Germany; 3 Institute of Biomechanics, TUHH Hamburg University of Technology, Hamburg, Germany; 4 Institute of Forensic Medicine, University Medical Center Hamburg-Eppendorf, Hamburg, Germany; 5 Department of Trauma Surgery, Orthopaedics and Sports Traumatology, BG Hospital Hamburg, Hamburg, Germany

**Keywords:** Shoulder surgery, Arthroscopy, Acromioclavicular joint, Suture button, Micro-CT, Tunnel widening

## Abstract

**Purpose:**

Biomechanical evaluation of three different suture button devices used in acromioclavicular joint repair and analysis of their effect on post-testing tunnel widening.

**Methods:**

Eighteen human shoulder girdles were assigned into three groups with a similar mean bone mineral density. Three different single-tunnel acromioclavicular repair devices were tested: (1) AC TightRope^®^ with FiberWire; (2) AC Dog Bone^™^ Button with FiberTape; (3) Low Profile AC Repair System. Biomechanical testing was performed simulating the complex movement of the distal clavicle as follows. A vertical load of 80 N was applied continuously. The rotation of the clavicle about its long axis was set at 10° anterior and 30° posterior for 2500 cycles at 0.25 Hz. The horizontal translation of the clavicle was set at 6 mm medial and 6 mm lateral for 10,000 cycles at 1 Hz. The coracoclavicular distance was measured before and after testing. After testing, each sample underwent micro-CT analysis. Following 3D reconstruction, the area of the bone tunnels was measured at five defined cross sections.

**Results:**

In TightRope^®^ and Dog Bone^™^ groups, all samples completed testing, whereas in the Low Profile group, three out of six samples showed system failure. The mean absolute difference of coracoclavicular distance after testing was significantly greater in the Low Profile group compared to TightRope^®^ and Dog Bone^™^ groups (4.3 ± 1.3 mm vs 1.9 ± 0.7 mm vs 1.9 ± 0.8 mm; *p* = 0.001). Micro-CT analysis of the specimens demonstrated significant tunnel widening in the inferior clavicular and superior coracoid regions in all three groups (*p* < 0.05).

**Conclusion:**

Significant tunnel widening can be observed for all devices and is primarily found in the inferior parts of the clavicle and superior parts of the coracoid. The Low Profile AC Repair System showed inferior biomechanical properties compared to the AC TightRope^®^ and AC Dog Bone^™^ devices. Therefore, clinicians should carefully select the type of acromioclavicular repair device used and need to consider tunnel widening as a complication.

## Introduction

Arthroscopically assisted AC (acromioclavicular) joint repair using synthetic augmentation techniques is well established [18, 21, 22, 31]. It is most commonly used in acute high-grade AC joint dislocations, yielding good clinical results [4, 18, 20–22, 31]. Most of the various methods are performed by drilling one or two transclavicular and transcoracoid tunnels through which the different devices are threaded. Usually, they are fastened in a pulley-like fashion and fixated using a suture button device to secure the clavicle in place, thereby restoring the AC joint anatomy [7, 11, 14, 18, 24]. Thus, it allows healing of the coracoclavicular ligaments in the previous position to achieve long-term stability [18]. However, loss of reduction appears to be a frequently observed complication, reported in 12–80% of cases [3, 7, 13, 19, 27]. Recently, the potential impact of tunnel width on the loss of reduction has gained further interest [2, 17, 23, 25, 28]. Several biomechanical studies have demonstrated that large tunnels are associated with an increased fracture risk and with reduced load to failure [6, 7, 10, 26]. Furthermore, in several studies, progressive tunnel widening was observed during the postoperative course, with differing results regarding loss of reduction [1, 2, 9, 16, 23, 25, 28]. Influencing factors might be the material and type of AC repair device and associated micro-motions between the bone and suture material as well as heat-induced osteolysis due to the bone drilling. This may contribute to a loss of reduction and an increased fracture risk over time [2, 7, 8, 23, 28, 29]. Recent implant development aims to reduce bone tunnel widening using an unicortical insert button (Low Profile AC Repair System) [5]. Still, this hypothesis has not been proven and the specific characteristics of postoperative tunnel widening remain unclear. Therefore, the purpose of this study was to biomechanically evaluate three different suture button devices and analyze their impact on potential tunnel widening. Furthermore, the properties of the tunnels were evaluated to gain further insight into their morphology post-widening. The clinical relevance of this study is to offer surgeons relevant information on which device to use and if tunnel widening needs to be considered in AC suture button repair. The hypothesis is that all devices lead to tunnel widening.

## Materials and methods

Approval was obtained from the institutional review board (Ethical Review Committee Hamburg, Germany; study number: WF-183/20). A total of 18 human shoulder girdles (en bloc resection of scapula and clavicle, nine matched pairs) were collected from donors aged 35–88 years (mean 61.6 ± 16.6 years). The donor specimens were acquired from the local Institute of Forensic Medicine [15]. After harvesting, the specimens were sealed in plastic bags and stored at a temperature below − 20 °C.

Each specimen was scanned using a 16-row CT scanner (Brilliance 16 CT; Philips Healthcare, Hamburg, Germany) with a solid calibration phantom (Bone Density Calibration Phantom; QRM, Moehrendorf, Germany) to determine the volumetric bone mineral density (vBMD) in terms of calcium hydroxylapatite (mg CaHA per cm^3^) (Avizo 5.1; VSG Inc., Burlington, Massachusetts) [12, 30]. According to the BMD, three groups (six samples each) with similar bone densities were formed.

On the day of testing, each thawed shoulder girdle was dissected as follows. All overlying soft tissue was removed to expose the bone surface of the clavicle and scapula. Preparation of the coracoclavicular (CC) region was performed with special care to prevent injury to the CC ligaments. A k-wire was placed laterally through the AC joint to secure the anatomic AC position. The scapula was horizontally transected 2 cm below the coracoid, the specimen was potted upright in a steel tube, and the caudal end was fixed using a methyl methacrylate solution (Technovit 4004; Hereaus Kulzer, Hanau, Germany). Fixation was performed up to 1 cm proximal to the coracoid process. Perpendicularly to the scapula, the medial third of the clavicular was fixated in a similar manner. After correct positioning and fixation of the specimen in the test rig, the lateral k-wire was removed and the AC joint capsular ligament as well as the CC ligaments were divided to simulate a high-grade AC joint disruption. Throughout preparation and testing, the specimens were wrapped in moist tissue to preserve the constitution of the tissue. The experiments were performed at room temperature in a normal room environment.

Three different, commercially available AC repair devices were tested: TightRope^®^: A double-button AC reconstruction system consisting of a four-strand continuous loop of multi-strand, long-chain ultra-high-molecular-weight polyethylene suture (UHMWPE), which is interlaced between two titanium cortical buttons. The inferior button is stick-shaped and needs to be shuttled through the CC bone tunnel before it is placed underneath the coracoid arch. The tunnel drill size of this device is 3.5 mm (AC TightRope^®^ with #5 FiberWire; Arthrex Inc., Naples, Florida, USA). Dog Bone^™^: a double-button AC reconstruction system consisting of a UHMWPE suture tape with a width of 2 mm which is attached independently to two dog-bone-shaped cortical titanium buttons. Therefore, just the tape needs to be passed through the coracoid and clavicle tunnels. The drill size is 3 mm (AC Dog Bone^™^ Button with 2 mm FiberTape; Arthrex Inc., Naples, Florida, USA). Low Profile: a double-button AC reconstruction system consisting of an inferior dog-bone-shaped button, similar to the AC Dog Bone^™^ button, and a superior clavicle titanium insert button which is inserted 5 mm unicortically into the clavicle. According to the manufacturer, this insert button might prevent suture abrasion. The sutures are similar to the ones used in the AC TightRope^®^ device and are tensioned with a suture tensioning instrument. According to the manufacturer’s instructions, the suture tensioning instrument should be used to check mark 50—which accounts to 220 N if compared to a load cell. During the first implantation with this tensioner, it was noticed that this force would be too high—especially taking into consideration that the recent literature advises a tensioning force of 80–120 N specifically for this device [5, 11]. Therefore, the tensioner was not used in the following samples. The sutures were manually fastened (as strongly as possible). The CC gap was preserved during fastening by a spacer. During implantation of the device and the subsequent in vitro testing, a vertical force of 80 N was applied by dead weights to account for the pull force of the trapezius muscle. The drill size of this device is 3 mm, but it has a 5.1 mm unicortical socket at the superior clavicle (insert button) (Low Profile AC Repair System; Arthrex Inc., Naples, Florida, USA).

Implantation of the AC reconstruction devices was performed according to the manufacturers’ instructions. Before definitive insertion of the implants, a 1 mm k-wire was temporarily placed at the correct CC position to ensure proper placement of all drilling holes.

**Fig. 1:**
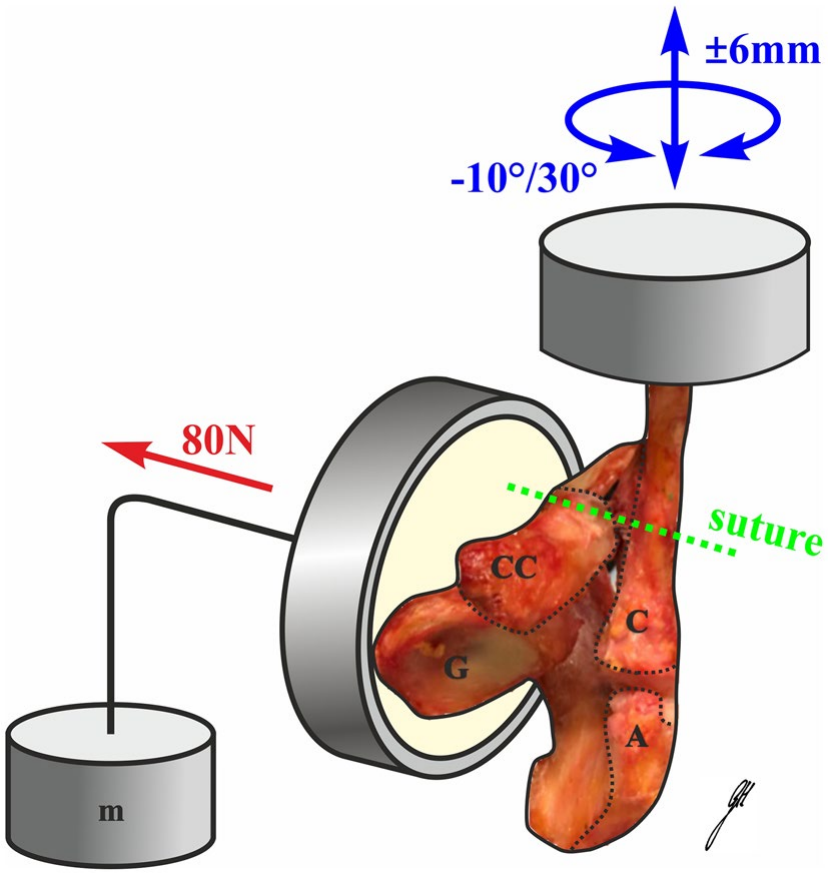
Experimental set-up. *C* clavicle, *CC* coracoid process, *G* glenoid, *A* acromion, *m* mass

The biomechanical set-up and protocol were performed as previously described by Kippe et al. consisting of cyclic axial rotation of the clavicle with a concomitantly applied vertical load [8]. Due to the alignment of the servo-hydraulic testing machine (MTS 858.2; MTS Systems, Eden Prairie, Minnesota, USA), the specimens were placed in a lateral position. In the following, the indications of direction are anatomically oriented for a human neutral standing position. A load cell six degrees of freedom (6DOF) was used (SN 30 031; Huppert, Herrenberg, Germany). Horizontal instability was simulated by rotating the clavicle about its long axis at 10° anterior and 30° posterior (total range 40°) for 2500 cycles at 0.25 Hz and a horizontal translation of the clavicle at 6 mm medial and 6 mm lateral (12 mm in total) for 10,000 cycles at 1 Hz. This movement mimics the AC overlap in the context of horizontal instability comparable to the mechanics of forced adduction of the shoulder. The 80 N vertical load was applied continuously using a dead weight (Fig. 1). The accuracy of the universal testing machine in its working range is denoted as class 0.5 for axial displacement and rotational angle.

**Fig. 2:**
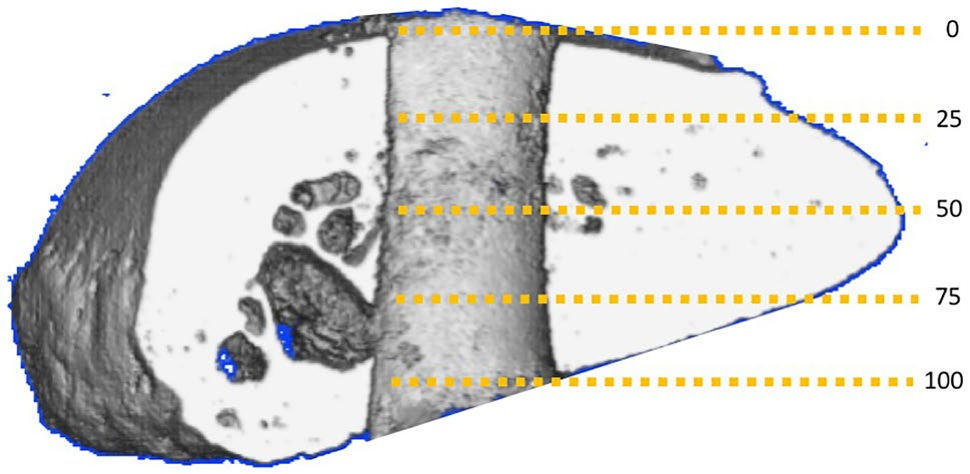
Micro-CT imaging of the post-testing tunnel in the distal clavicle including the five defined measurement points: superior (0) and inferior cortex (100), as well as at 25%, 50%, and 75% of the total vertical bone diameter

The CC distance (distance between the superior cortex of the coracoid and the inferior cortex of the clavicle) was manually measured before and after testing. Construct failure was defined as a CC displacement of > 10 mm. This protocol was designed, because it includes the physiological multidimensional movement of the lateral clavicle by combining axial rotation with a concomitantly applied vertical load as well as horizontal translation. Following biomechanical testing, each sample underwent micro-CT analysis (μCT 35, 30–70 kV; Fa. Scanco Medical, Wangen-Brüttisellen, Switzerland). Before analysis, the implants were carefully removed and an additional drilling hole with the same diameter as that for each device was drilled next to the tunnel to serve as control group (*n *= 4 in each group). After automatic 3D reconstruction of the region of interest (Avizo evaluation program; Thermo Fisher Scientific, Waltham, Massachusetts, USA), the areas of the transversal plane of the coracoid and clavicular bone tunnels, as well as that of the control group, were calculated at five defined points each: superior (0) and inferior cortex (100) of the coracoid or clavicle, as well as at 25%, 50%, and 75% of the total vertical bone diameter (Fig. 2). Measurements were performed at high resolution with a voxel size of 15 μm. A threshold of 900 was used to optimize the image.

### Statistical analysis

Two-factor ANOVA was performed with a significance level of *α* = 0.05 (SPSS, Version 21, IBM, Armonk, New York, USA). Analysis was performed separately at each of the ten measurement points for the factors device type, control group, and post-testing widening. Levene’s test was performed to further assess the equality of error variances.

## Results

### Bone mineral density

Determination of vBMD revealed similar values in all three groups [180 ± 8 mg/cm^3^ in the TightRope^®^ group (*n *= 6), 180 ± 10 mg/cm^3^ in the Dog Bone™ group (*n *= 6) and 180 ± 7 mg/cm^3^ in the Low Profile group (*n *= 6)].

### Coracoclavicular distance

In the TightRope^®^ (*n *= 6; AC TightRope^®^ with FiberWire) and Dog Bone^™^ (*n *= 6; AC Dog Bone^™^ with FiberTape) groups, all samples completed biomechanical testing without failure. In the Low Profile group, three out of six samples showed a post-testing difference of > 10 mm and were therefore categorized as system failure. The Low Profile group showed a significantly elongated CC distance compared to TightRope^®^ and Dog Bone^™^ groups (*p* = 0.001). The mean absolute difference of CC distance for each device is displayed in Table 1.

**Table 1:** Mean absolute difference of coracoclavicular distance after testing for each device

AC repair device	Coracoclavicular distance (mm)
TightRope^®^	1.9 ± 0.7
Dog Bone^™^	1.9 ± 0.8
Low Profile	4.2 ± 1.3*

*Indicates statistical significance with *p* = 0.001

### Tunnel width

**Fig. 3:**
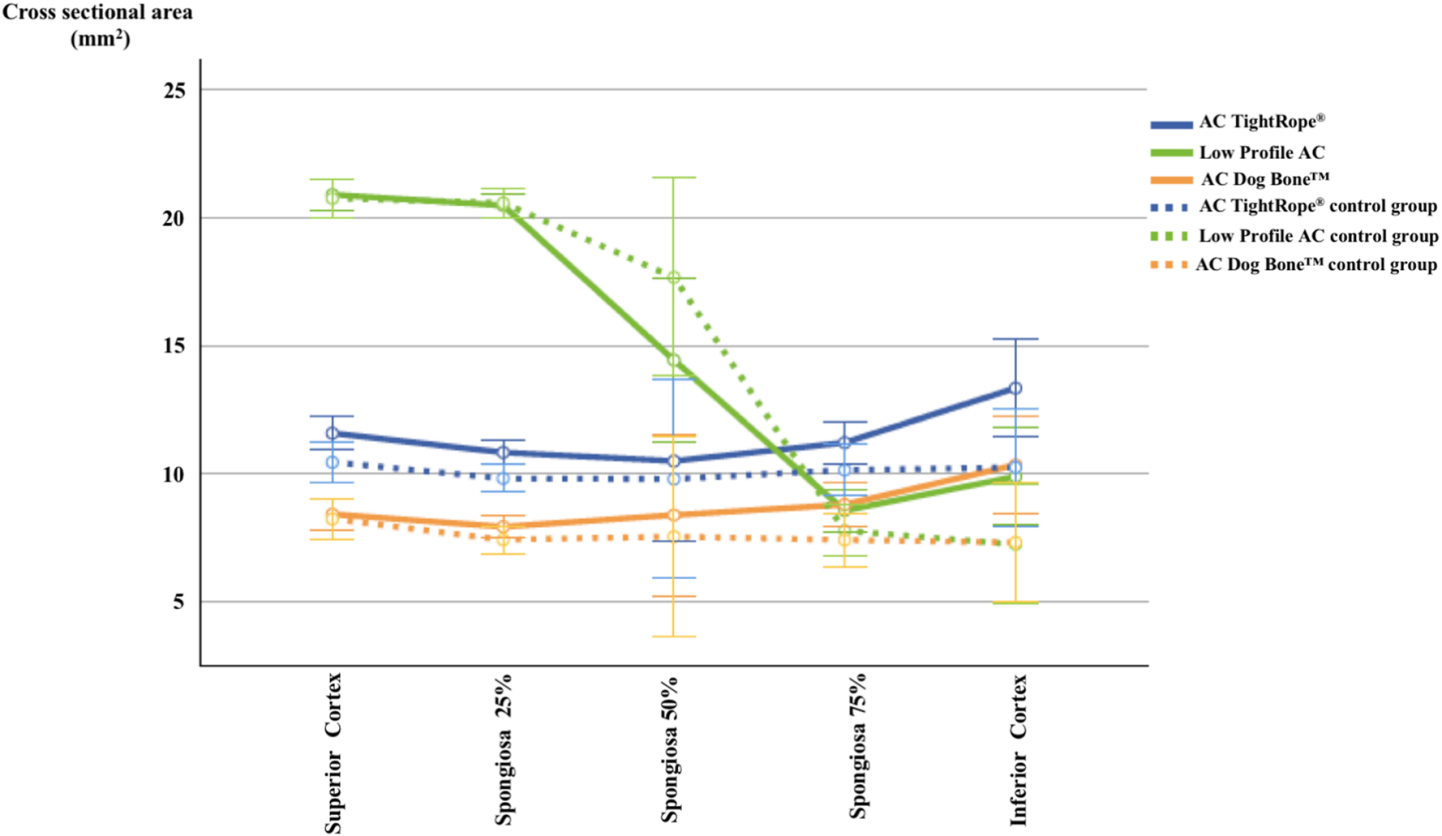
Clavicular tunnel widening in TightRope^®^, Dog Bone^™^, and Low Profile groups at each defined measurement point in comparison to each control group. Tunnel widening occurred in all three devices at the inferior clavicular cortex as well as at 75% spongiosa. Differences in tunnel width at measurement point 50% spongiosa are related to the changing drill diameter of the insert button in relation to the diameter of the clavicula

**Fig. 4:**
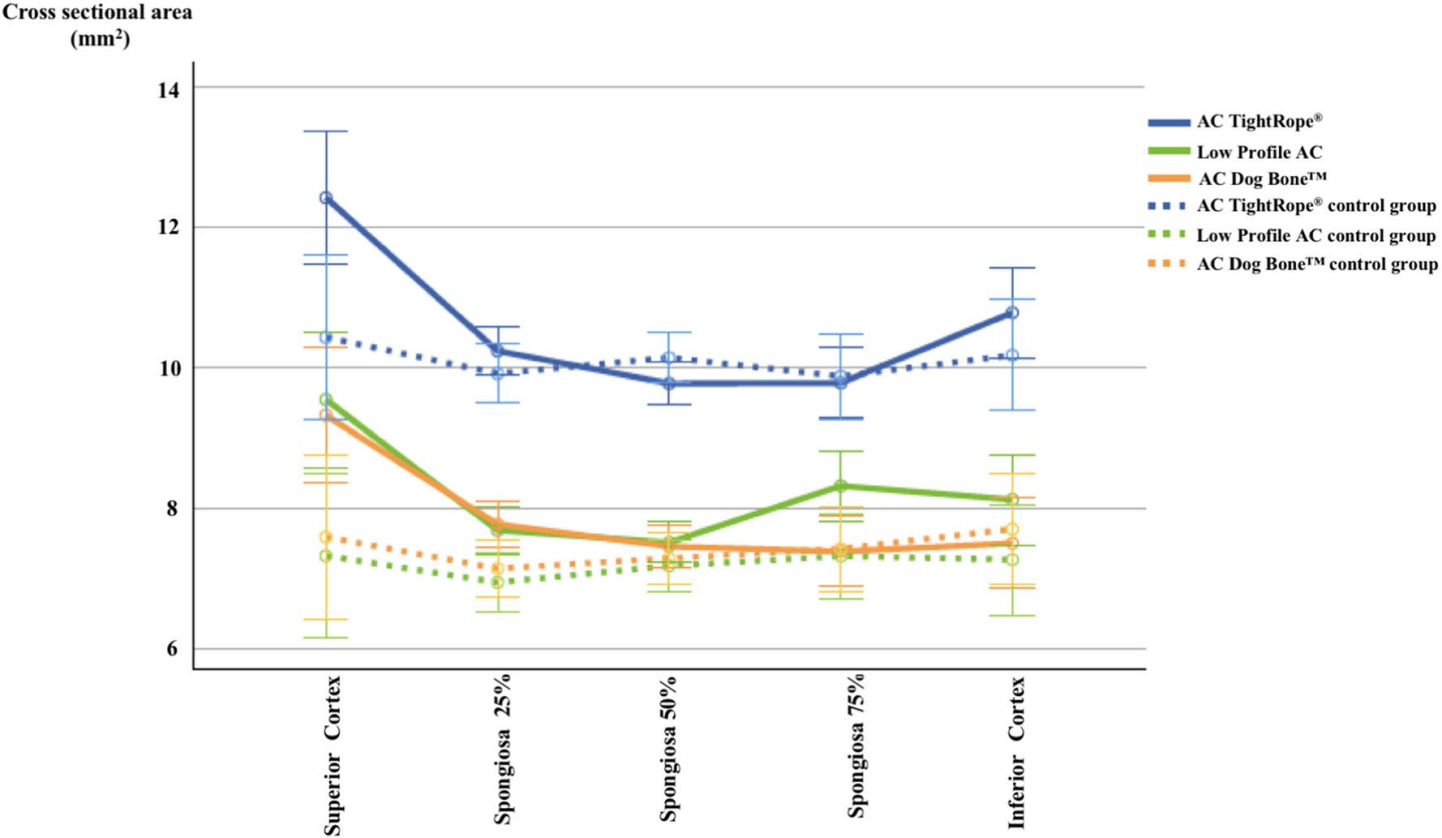
Coracoid tunnel widening in TightRope^®^, Dog Bone^™^, and Low Profile groups at each defined measurement point in comparison to each control group. Tunnel widening occurred in all three devices at the superior cortex as well as at 25% spongiosa

Analysis of post-testing tunnel width revealed significant tunnel widening for all three devices compared to the control group. In all three groups, tunnel widening occurred in the inferior clavicular and superior coracoid cortex as well as at 75% spongiosa of the clavicle and 25% spongiosa of the coracoid (Figs. 3 and 4; Table 2).

**Table 2:** *P* values for tunnel widening at significant measurement points

Bone	Measurement point	Levene	Post-testing	Type	Interaction
Clavicle	75% Spongiosa	0.09	0.007	< 0.001	0.81
	Inferior cortex	0.08	0.002	0.007	0.97
Coracoid	Superior cortex	< 0.001	< 0.001	< 0.001	0.89
	25% Spongiosa	0.94	0.001	< 0.001	0.51

Post-testing represents the difference of the control group compared to the bone tunnel after testing. Type represents the device-specific difference. Interaction represents the differences in tunnel widening in between the used devices

The maximum tunnel enlargement was detected in the inferior clavicular cortex, with a relative increase of 30% in the TightRope^®^ group, 41% in the Dog Bone^™^ group and 36.5% in the Low Profile group, followed by that in the superior cortex of the coracoid process, with a relative increase of 19% (TightRope^®^), 22% (Dog Bone^™^) and 30% (Low Profile). Comparing the different groups to each other (interaction), no significant difference could be detected regarding tunnel widening at any measurement point (Table 2).

## Discussion

The most important finding of the present study was that tunnel widening occurs after AC joint reconstruction in all three double-button systems tested. In particular, tunnel widening was observed in the inferior part of the clavicle and the superior part of the coracoid process. The Low Profile AC Repair device with a specifically designed insert button showed significantly higher failure rates compared to the two conventional double-button systems.

While several studies have shown that an increased diameter and number of initial drill holes are associated with an increased risk of coracoid process and clavicle fracture, more recently, the potential progressive tunnel widening during the postoperative course is gaining more attention [6, 7, 10, 17, 24, 26]. In studies focusing on this phenomenon, tunnel widening could be observed in 55–100% of the patients, with a relative increase in tunnel diameter of 17.9–66.6% [1–3, 9, 16, 23, 25, 28]. Similar values were reached in this study, with a relative increase in tunnel width of up to 41%. Still, comparison is limited, since most studies determined the bone tunnel in a postoperative clinical setting via measurements on an anterior–posterior radiograph, while this study analyzed tunnel widening via micro-CT scan after cyclic biomechanical testing. The results of this study demonstrate that tunnel widening occurs in the inferior part of the clavicle and superior part of the coracoid and not homogeneously throughout the whole drilling tunnel. Using a similar double-button AC TightRope^®^ device, Kraus et al. observed similar results for the clavicle bone tunnel, with increasing cone-shaped widening from superior to inferior parts [9]. This type of tunnel widening might be explained through micro-motions of the suture device comparable to a three-dimensional windshield-wiper effect, which would explain the maximum enlargement occurring as far distally from each fixpoint (button–cortex interface) as possible. Furthermore, it might be possible that this observed localization of tunnel widening symbolizes the starting point of the progressive widening phenomenon. Discrepancies in tunnel widening between studies might furthermore depend on the device used for AC repair, the angle of implant insertion, the time point of measurement as well as the method of measurement [2, 23]. Berthold et al. suggested that biological tendon graft augmentation, frequently used in chronic AC dislocations, induces the windshield-wiper effect, while high-tensile suture devices rather cut into the bone [2]. The three-dimensional bone tunnel reconstruction via micro-CT in this study contradicts this assumption, since a suture cutting into the bone could not be observed using this set-up. Moreover, this study could not find a difference in tunnel widening between the differently shaped suture devices (tape and wire). To prevent tunnel widening due to frictional damage of the high-tensile sutures, a new Low Profile AC Repair System device was recently introduced in the context of acute and chronic AC joint instability [5, 11]. Based on the results of this study, this device and its superior clavicle insert button which is inserted 5 mm unicortically in the superior clavicle does not inhibit tunnel widening, since the origin of clavicular tunnel widening was observed in the inferior parts. Moreover, three out of six samples failed before cyclic testing was completed, and the mean CC distance of the remaining samples after testing was 4.2 mm compared to 1.9 mm for the two other devices. Furthermore, due to the comparably large unicortical drill hole needed, pronounced bone damage needs to be considered as well. Therefore, based on these biomechanical data, usage of this device cannot be recommended. The reason for the early failure might be potential suture abrasion at the edge of the unicortical button or an insufficient locking mechanism of the insert button. This potential suture abrasion might be especially due to horizontal instability. Further studies have to prove if, e.g., an additional cerclage fixation of the AC joint might lower the risk of failure in this device. In this biomechanical study, no correlation between CC elongation or failure rate and tunnel widening could be detected, therefore questioning the impact of tunnel widening and supporting recent clinical results for which no relationship could be established between tunnel widening and loss of reduction or clinical outcome [2, 28]. Still, progressive tunnel widening over time might increase the fracture risk and therefore indirectly be a clinical risk factor.

The limitations of this study include the in vitro set-up and the relatively high age of the donors. Direct clinical translation is therefore limited. Furthermore, the availability of human shoulder girdles for in vitro testing is restricted and this limitation in sample size also limits the power to accept the null hypotheses. Also, experiments were performed using three specific suture button systems with a single-tunnel technique. Due to the wide range of available techniques and devices, a general conclusion cannot be finally drawn. This biomechanical set-up induces horizontal instability. Thus, it might be that an additional AC cerclage would influence tunnel widening as well. Still, in clinical studies where an additional cerclage was performed, tunnel widening nevertheless occurred [28]. Furthermore, it needs to be noticed that the tensioning instrument for the Low Profile AC implantation was not used in five out of six samples. Therefore, the higher failure rates for this device need to be carefully interpreted. However, recent literature using this reconstruction system recommends a tension load of 80–120 N [5, 11] which was assured in this study. It also needs to be recognized that the Levene’s test disproved the equality of error variances for three measurement points (50% spongiosa of the clavicula as well as superior and inferior cortex of the coracoid). Those values have to be taken with care, but are reported for the comparison.

Overall, the clinical relevance of this study is that clinicians should carefully select the type of AC repair device used and need to consider tunnel widening as a complication.

## Conclusion

The present study demonstrates significantly higher failure rates for the new Low Profile AC Repair System device compared to the conventional double-button devices. Tunnel widening was observed for all three devices and was located in the inferior parts of the clavicle and superior parts of the coracoid. Therefore, new devices should include amendments in these specific regions to avoid tunnel widening.
